# Supplementary material for: Tree species traits affect which natural enemies drive the Janzen-Connell effect in a temperate forest
Source: Nat Commun. 2020 Jan 15;11:286. doi: 10.1038/s41467-019-14140-y (PMC6962457; doi:10.1038/s41467-019-14140-y)
Supplement: Supplementary file 1 — Supplementary Information File [file 41467_2019_14140_MOESM1_ESM.pdf]

Supplementary Information

Tree species traits affect which natural enemies drive the Janzen-Connell effect in a temperate forest

Jia et al.

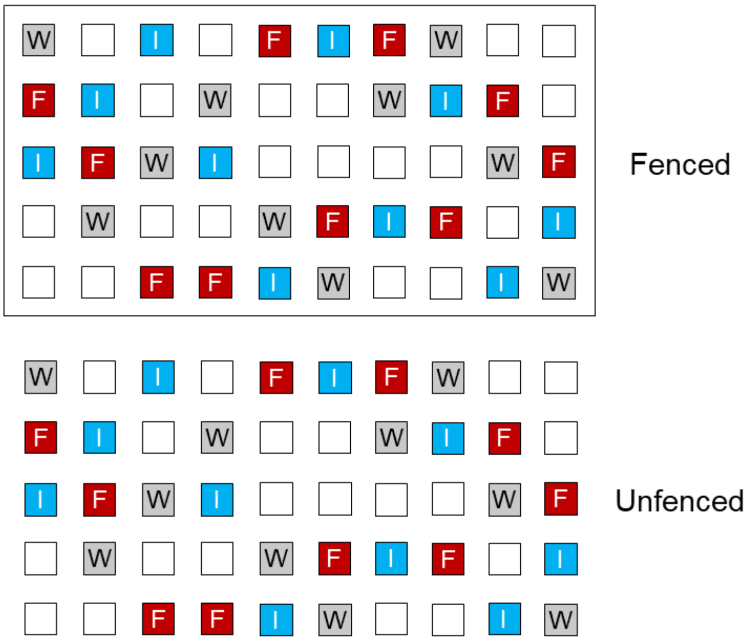

Supplementary Figure 1. The experimental layout. The three 55 × 50 m blocks, each containing a fenced and unfenced plot, were located at least 200 m from each other. Within each plot, ten 1 ×1 m^2 quadrats were randomly allocated to each of the following treatments: fungicide (F), insecticide (I), water (control of pesticide treatment; W).

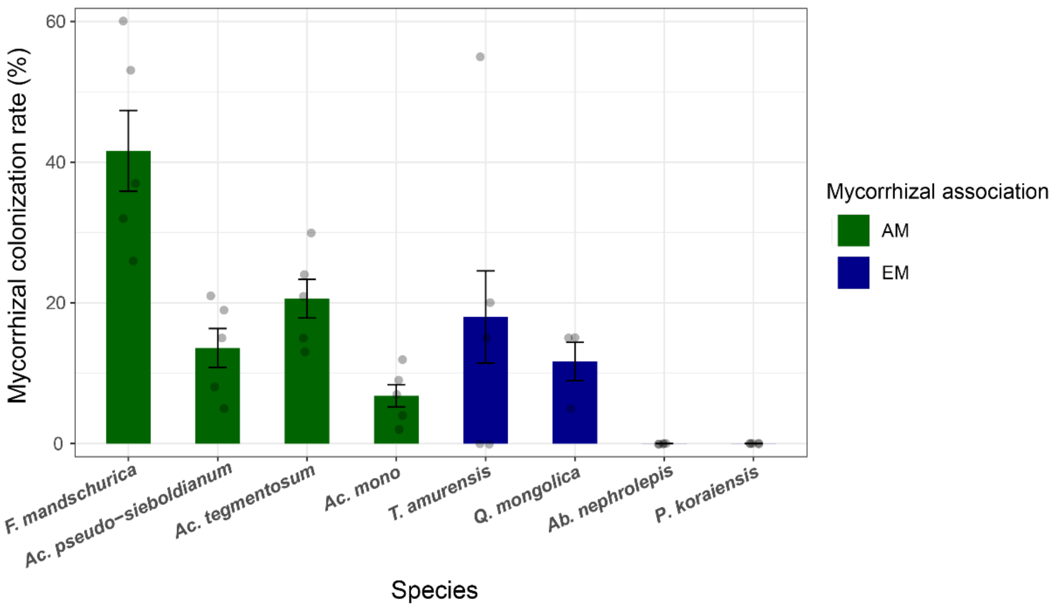

Supplementary Figure 2. Mycorrhizal colonization (mean ± SE) of tree seedling roots. Each point represents an individual measurement for each seedling. Roots of eight abundant tree species were examined for mycorrhizae (four EM species: *T. amurensis*, *Q. mongolica*, *Ab. nephrolepis*, and *P. koraiensis*; and four AM species: *F. mandschurica*, *Ac. pseudo-sieboldianum*, *Ac. mono and Ac. tegmentosum*). For each species, we randomly selected five seedlings to measure root colonization, except for *Q. mongolica* (n = 3) that had very low recruitment in the year of sampling. Roots of current-year seedlings were harvested within or near our experimental blocks in late September 2019. For each seedling, 20 first-order roots were dissected from root-branching system and used for determining mycorrhizal colonization. All first-order roots were cleared in 10% KOH at 90°C for 2-5 h depending on tree species, acidified with 5% HCl for 5 min, stained in 0.05% trypan blue for 12 h, and destained in solution of water-glycerol-acetic acid (1:1:1) for 48 h. EM colonization rate was determined using a compound light microscope (100× magnification; Olympus CX33, Tokyo, Japan) as the percentage of first-order roots colonized by EM fungi, which are characterized by the presence of the fungal mantle and hartig net. AM colonization rate was quantified using a compound light microscope (200 × magnification) based on the magnified intersection method[1].

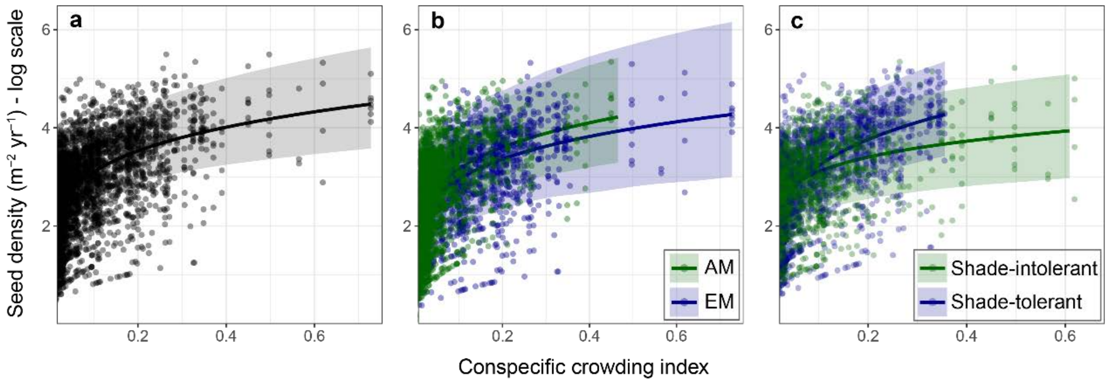

Supplementary Figure 3. Seed density near conspecific adults. The relationship between seed density and conspecific adult density for **a** overall, and **b, c** different type of mycorrhizal association and shade tolerance, respectively. Given that we did not install seed traps in our experimental blocks, the data used here are from the Changbaishan (CBS) plot, 200 ~ 500 m away from any of our experimental blocks. Seed traps were established in the CBS plot following the field protocol of the Center for Tropical Forest Science (CTFS) of the Smithsonian Institution. Seeds were collected from traps twice per month from May to December and once per month for the rest of the year. Damaged and immature seeds were discarded and the remaining seeds counted and identified to species. Seed data from 2010 to 2017 were used in this analysis. The conspecific adult density (i.e. conspecific crowding index) was calculated by summing the inverse-distance weighted basal area of all trees > 5 cm diameter at breast height (DBH) within a 20 m radius of each seed trap. Seed density is plotted in log scale to facilitate visualization. Lines are the relationships fitted with generalized linear mixed-effects models with negative binomial error distribution, with means and 95% confidence intervals. Dots represent the observed seed densities, which were calculated by adding model residuals to the predicted values.

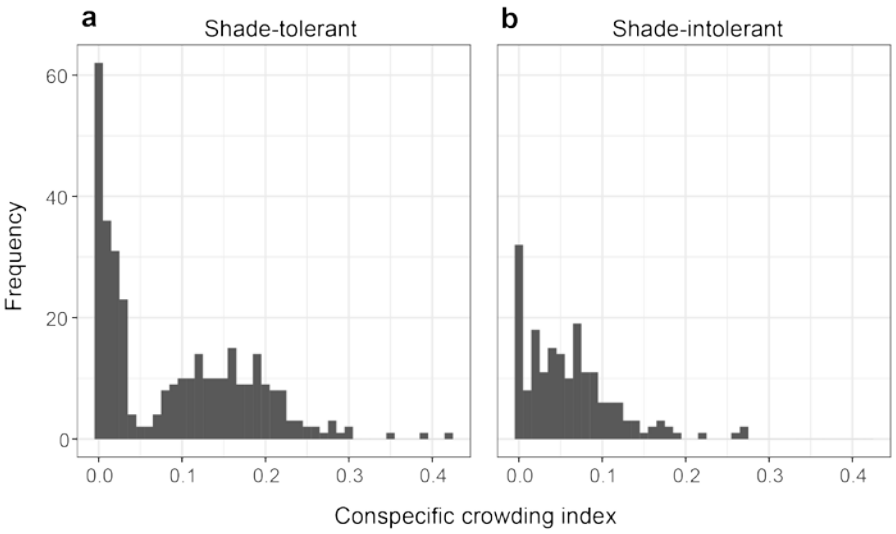

Supplementary Figure 4. The frequency distributions of conspecific adult density (i.e. conspecific crowding index) for different type of shade-tolerance in our experimental plots. **a** shade-tolerant, and **b** shade-intolerant species.

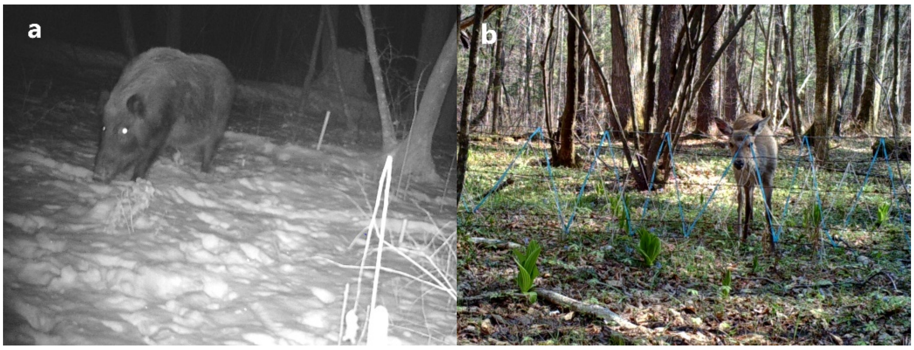

Supplementary Figure 5. The main large mammals captured by camara traps outside the fences. **a** Wild boar, and **b** Roe deer. Credit: S. Jia.

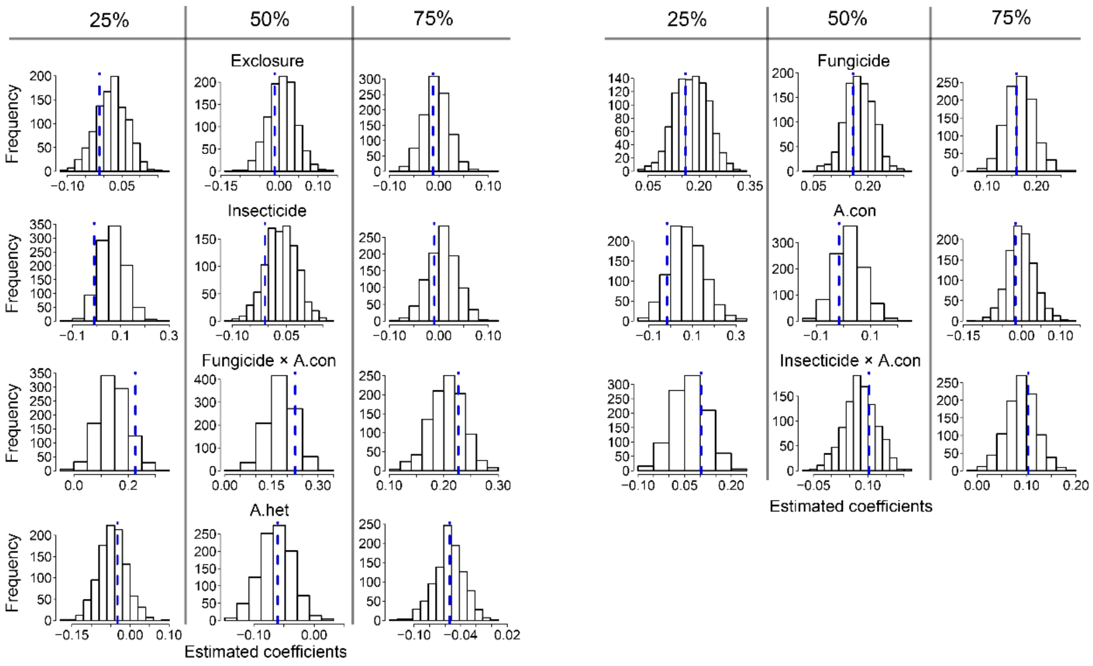

Supplementary Figure 6. Sensitivity of the community-wide results for the seedling recruitment analysis to random thinning of the two most abundant tree species. Generalized linear mixed effects models (GLMM) were fitted to estimate the effects of conspecific adult density (conspecific crowding index: A.con), pesticide treatment and their two-way interaction on seedling recruitment (for details see the legend of Figure 1). Frequency distributions of parameter estimates from GLMM are presented after randomly removing 25%, 50% and 75% of seedlings of *T. amurensis* and *F. mandschurica* from the whole dataset and repeating this procedure 999 times. Parameter estimates from these simulations were compared to the model including all seedlings (blue dashed lines).

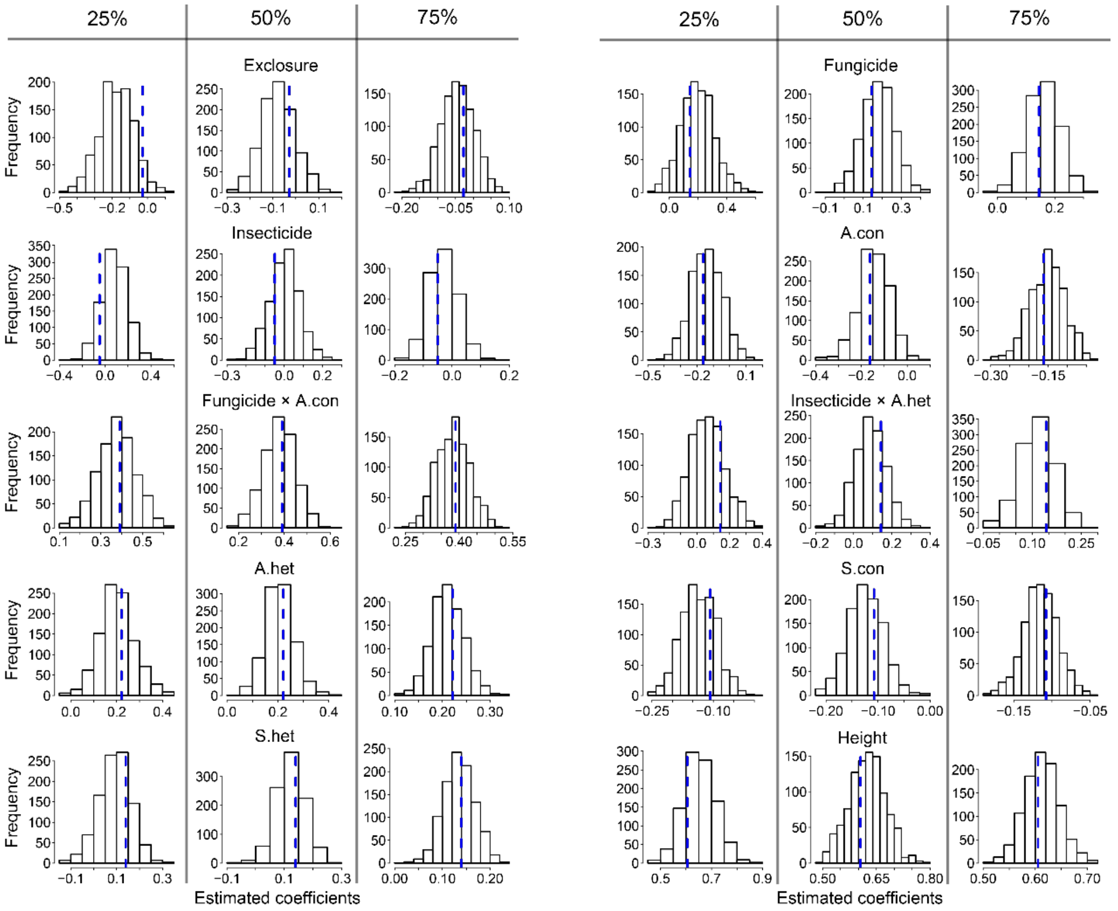

Supplementary Figure 7. Sensitivity of community-wide results of the seedling survival analysis to random thinning of the two most abundant tree species. Generalized linear mixed effects models (GLMM) were fitted to estimate the effects of conspecific adult density (conspecific crowding index: A.con), pesticide treatment and their two-way interaction on seedling survival. Frequency distributions of parameter estimates from GLMM are presented after randomly removing 25%, 50% and 75% of seedlings of *T. amurensis* and *F. mandschurica* from the whole dataset and repeating this procedure 999 times. Parameter estimates from these simulations were compared to the model including all seedlings (blue dashed lines).

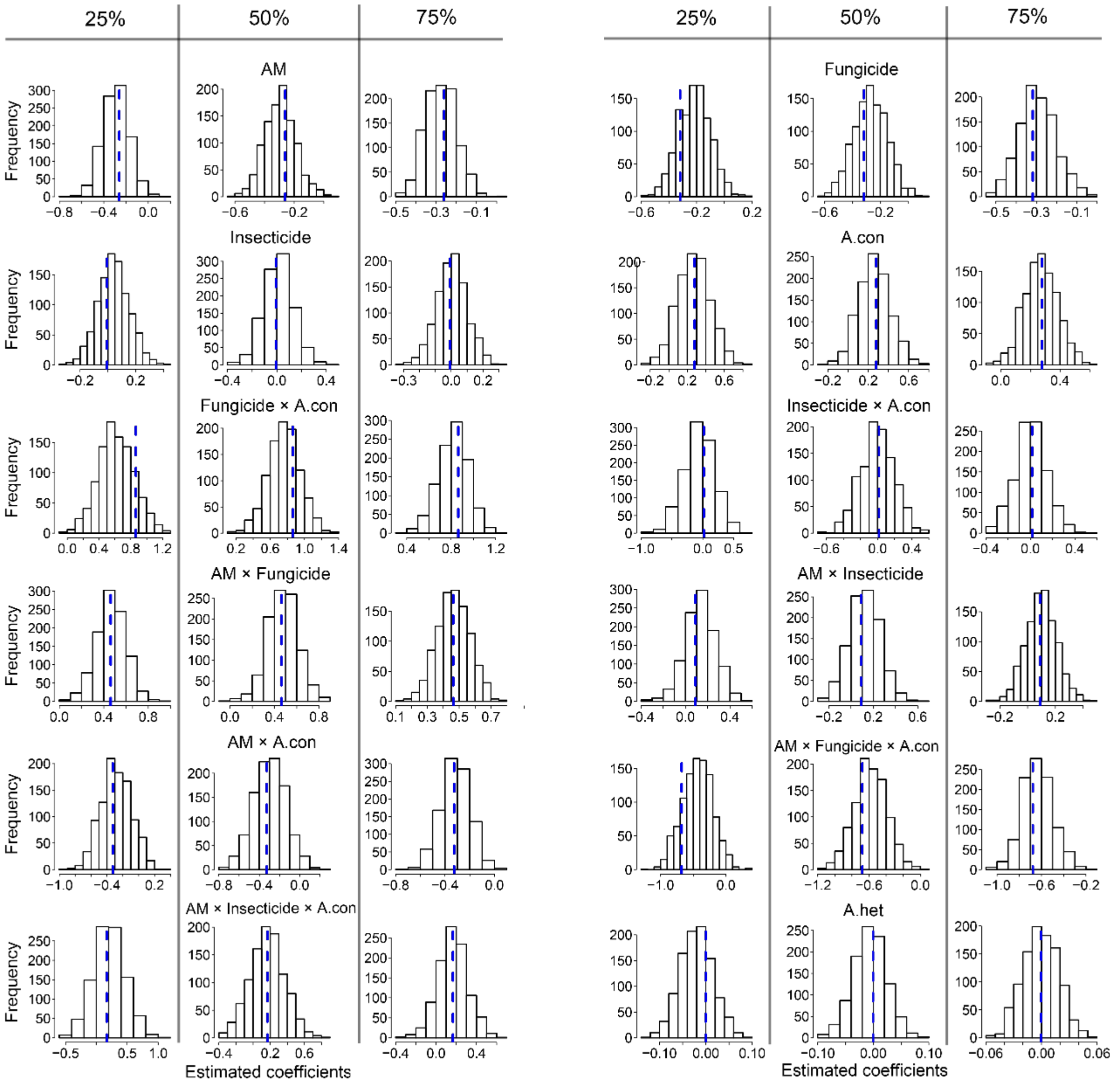

Supplementary Figure 8. Sensitivity of species' mycorrhizal association-seedling recruitment analyses to random thinning of the two most abundant tree species. Generalized linear mixed effects models (GLMM) were fitted to estimate the effects of conspecific adult density (conspecific crowding index: A.con), pesticide treatment, type of mycorrhizal association, and their two- and three-way interactions on seedling recruitment. Frequency distributions of parameter estimates from GLMM are presented after randomly removing 25%, 50% and 75% of seedlings of *T. amurensis* and *F. mandschurica* from the whole dataset and repeating this procedure 999 times. Parameter estimates from these simulations were compared to the models including all seedlings (blue dashed lines).

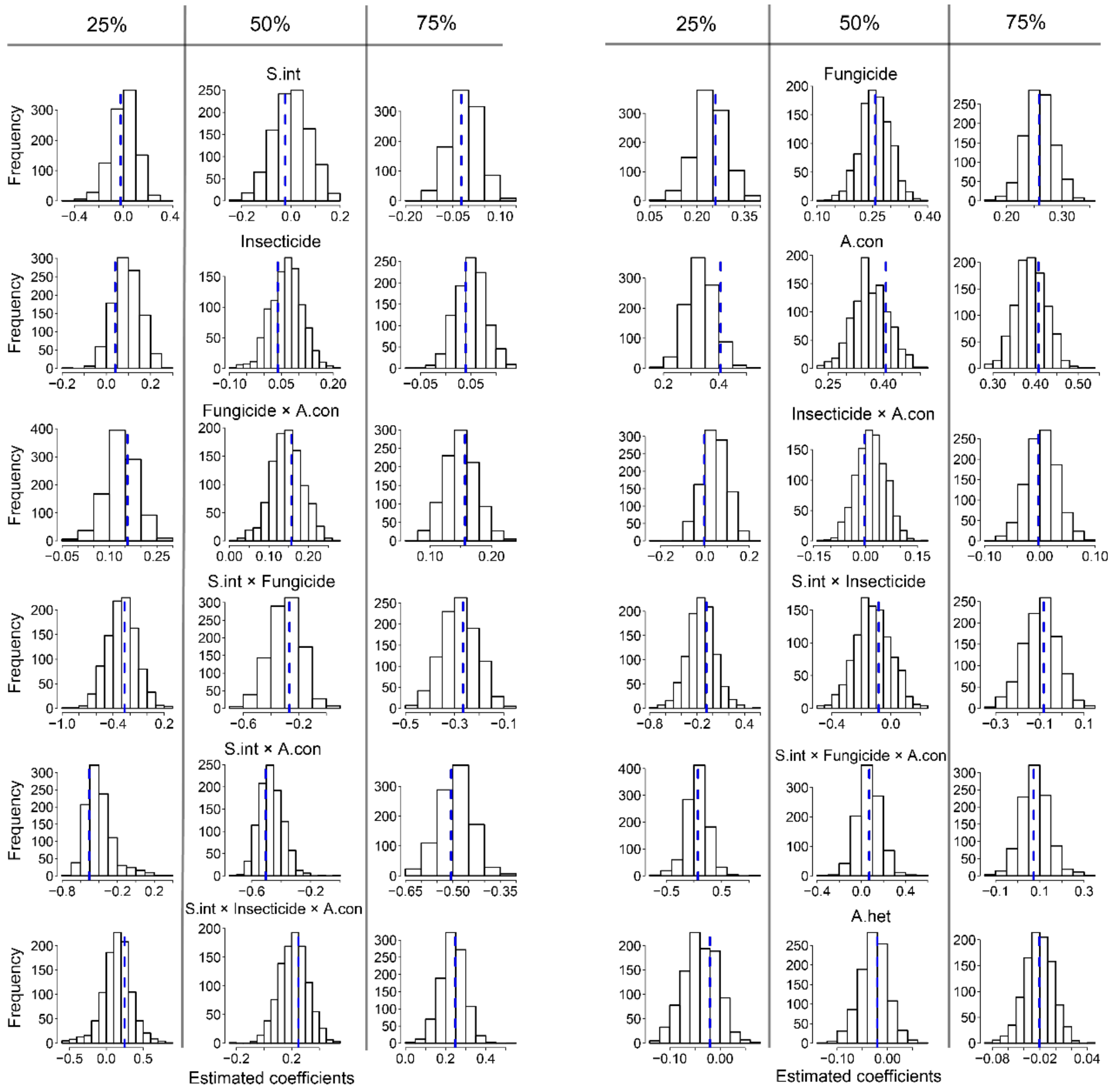

Supplementary Figure 9. Sensitivity of species' shade tolerance-seedling recruitment analyses to random thinning of the two most abundant tree species. Generalized linear mixed effects models (GLMM) were fitted to estimate the effects of conspecific adult density (conspecific crowding index: A.con), pesticide treatment, type of mycorrhizal association, and their two- and three-way interactions on seedling recruitment. Frequency distributions of parameter estimates from GLMM are presented after randomly removing 25%, 50% and 75% of seedlings of *T. amurensis* and *F. mandschurica* from the whole dataset and repeating this procedure 999 times. Parameter estimates from these simulations were compared to the models including all seedlings (blue dashed lines).

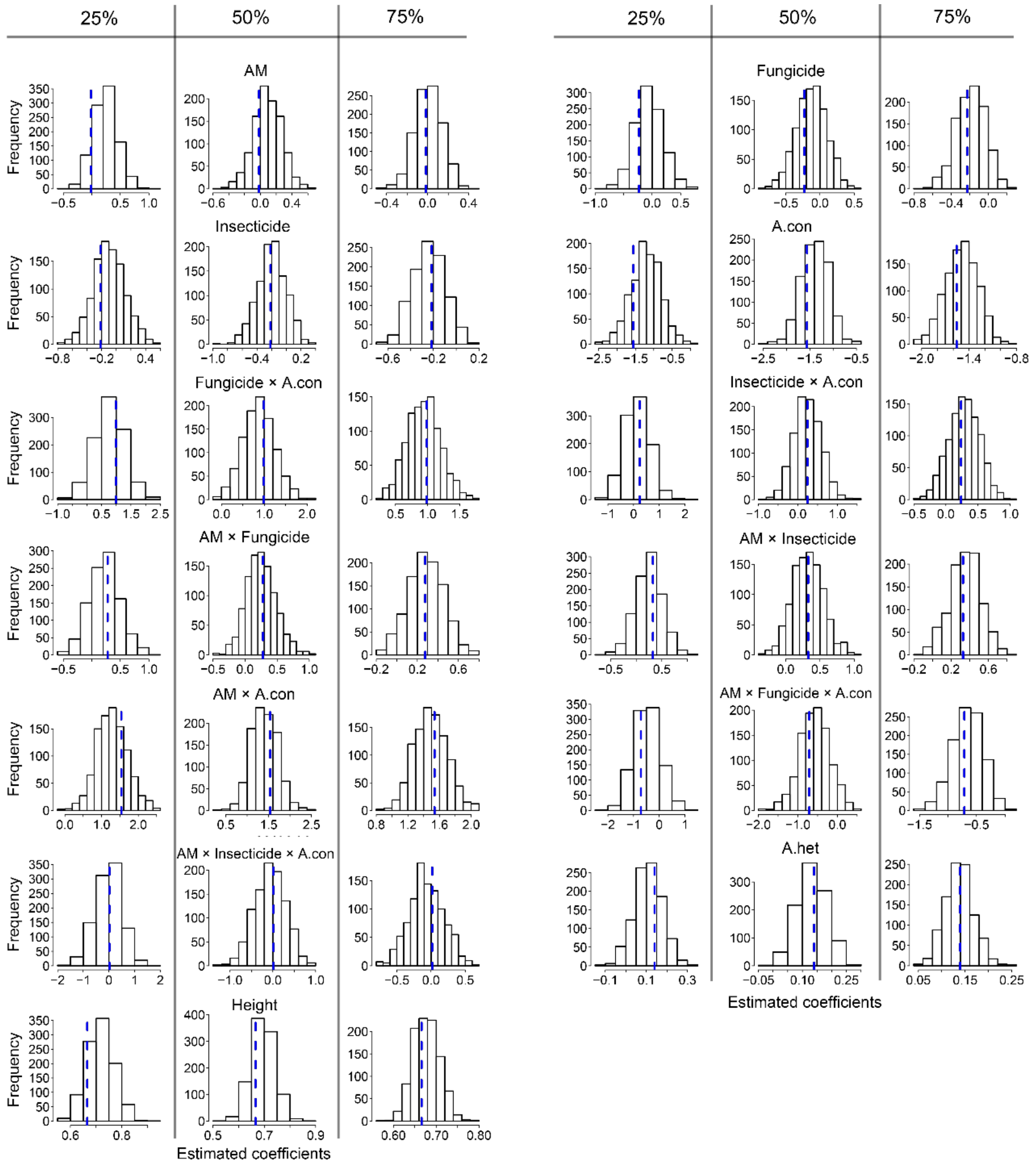

Supplementary Figure 10. Sensitivity of the species' mycorrhizal association-seedling survival analysis to random thinning of the two most abundant tree species. Generalized linear mixed effects models (GLMM) were fitted to estimate the effects of conspecific adult density (conspecific crowding index: A.con), pesticide treatment, type of mycorrhizal association, and their two- and three-way interactions on seedling survival. Frequency distributions of parameter estimates from GLMM are presented after randomly removing 25%, 50% and 75% of seedlings of *T. amurensis* and *F. mandschurica* from the whole dataset and repeating this procedure 999 times. Parameter estimates from these simulations were compared to the models including all seedlings (blue dashed lines).

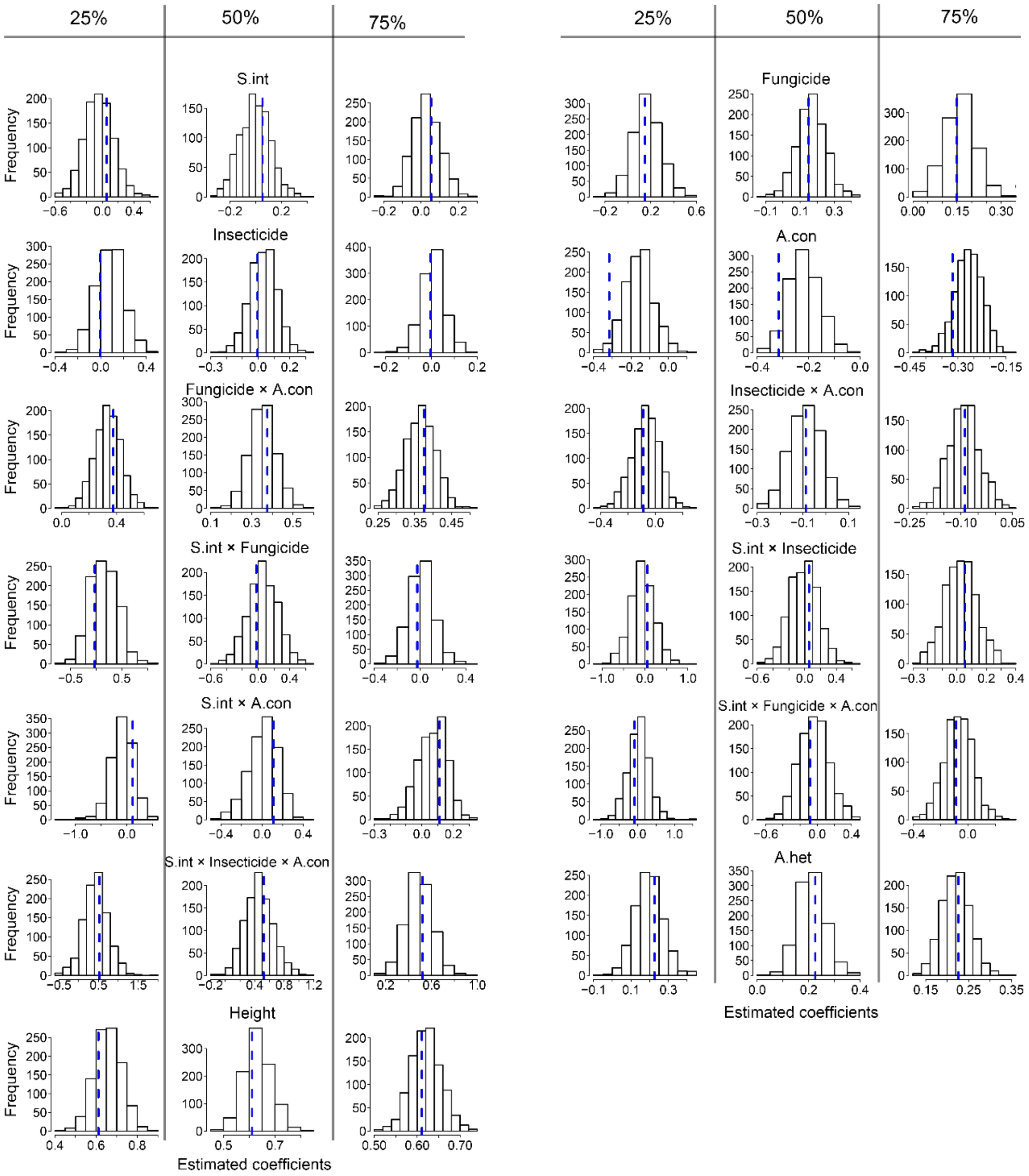

Supplementary Figure 11. Sensitivity of the species' shade tolerance-seedling survival analysis to random thinning of the two most abundant tree species. Generalized linear mixed effects models (GLMM) were fitted to estimate the effects of conspecific adult density (conspecific crowding index: A.con), pesticide treatment, shade tolerance status and their two- and three-way interactions on seedling survival. Frequency distributions of parameter estimates from GLMM are presented after randomly removing 25%, 50% and 75% of seedlings of *T. amurensis* and *F. mandschurica* from the whole dataset and repeating this procedure 999 times. Parameter estimates from these simulations were compared to the models including all seedlings (blue dashed lines).

Supplementary Table 1. Summary table of seedlings of tree species. Species are ranked in descending order of total number of seedlings.

Species	Family	Mycorrhizal association*	Shade tolerance†	Number of Seedlings
Tilia amurensis	Tiliaceae	EM	Shade tolerant	1688
Fraxinus mandschurica	Oleaceae	AM	Shade intolerant	1237
Acer pseudo-sieboldianum	Aceraceae	AM	Shade tolerant	387
Acer barbinerve	Aceraceae	AM	Shade tolerant	189
Abies nephrolepis	Pinaceae	EM	Shade tolerant	92
Acer tegmentosum	Aceraceae	AM	Shade tolerant	84
Pinus koraiensis	Pinaceae	EM	Shade intolerant	74
Acer mono	Aceraceae	AM	Shade tolerant	77
Quercus mongolica	Fagaceae	EM	Shade intolerant	69
Acer mandshuricum	Aceraceae	AM	Shade tolerant	17
Maackia amurensis	Leguminosae	AM	Shade intolerant	4
Ulmus japonica	Ulmaceae	AM	Shade intolerant	4
Tilia mandshurica	Tiliaceae	EM	Shade tolerant	3
Acer triflorum	Aceraceae	AM	Shade tolerant	2
Malus baccata	Rosaceae	AM	Shade tolerant	1
Phellodendron amurense	Rutaceae	AM	Shade intolerant	1

* Mycorrhizal association and † Shade tolerance were categorized following the criteria (refs. 2, 3), respectively.

Supplementary References

1. McGonigle, T. P., Miller, M. H., Evans, D. G., Fairchild, G. L. & Swan, J. A. A new method which gives an objective measure of colonization of roots by vesicular—arbuscular mycorrhizal fungi. *New Phytologist* **115**, 495–501 (1990).
2. Mao, Z. *et al.* Tree mycorrhizal associations mediate soil fertility effects on forest community structure in a temperate forest. *New Phytologist* (2019).
3. Wang, X. *et al.* Tree size distributions in an old-growth temperate forest. *Oikos* **118**, 25–36 (2009).
