# Supplementary material for: Tree species traits affect which natural enemies drive the Janzen-Connell effect in a temperate forest
Source: Nat Commun. 2020 Jan 15;11:286. doi: 10.1038/s41467-019-14140-y (PMC6962457; doi:10.1038/s41467-019-14140-y)
Supplement: Supplementary file 3 — Reporting Summary [file 41467_2019_14140_MOESM3_ESM.pdf]

nature research

Corresponding author(s): DBPR NCOMMS-19-20717B

Last updated by author(s): Nov 30, 2019

Reporting Summary

Nature Research wishes to improve the reproducibility of the work that we publish. This form provides structure for consistency and transparency in reporting. For further information on Nature Research policies, seeAuthors & Referees and theEditorial Policy Checklist .

Statistics

For all statistical analyses, confirm that the following items are present in the figure legend, table legend, main text, or Methods section.

n/a	Confirmed	
☐	☒	The exact sample size (n) for each experimental group/condition, given as a discrete number and unit of measurement
☐	☒	A statement on whether measurements were taken from distinct samples or whether the same sample was measured repeatedly
☐	☒	The statistical test(s) used AND whether they are one- or two-sided<br>*Only common tests should be described solely by name; describe more complex techniques in the Methods section.*
☐	☒	A description of all covariates tested
☐	☒	A description of any assumptions or corrections, such as tests of normality and adjustment for multiple comparisons
☐	☒	A full description of the statistical parameters including central tendency (e.g. means) or other basic estimates (e.g. regression coefficient) AND variation (e.g. standard deviation) or associated estimates of uncertainty (e.g. confidence intervals)
☐	☒	For null hypothesis testing, the test statistic (e.g. F, t, r) with confidence intervals, effect sizes, degrees of freedom and P value noted<br>*Give P values as exact values whenever suitable.*
☒	☐	For Bayesian analysis, information on the choice of priors and Markov chain Monte Carlo settings
☐	☒	For hierarchical and complex designs, identification of the appropriate level for tests and full reporting of outcomes
☒	☐	Estimates of effect sizes (e.g. Cohen's d, Pearson's r), indicating how they were calculated

Our web collection on statistics for biologists contains articles on many of the points above.

Software and code

Policy information about availability of computer code

Data collection	No software or code was used in the collection of data in this study. All analyses were based on previously collected experimental data.
Data analysis	All the data analyses were conducted using R (v 3.4.2) <http://www.R-project.org> using the package "lme4" (v 1.1.17).

For manuscripts utilizing custom algorithms or software that are central to the research but not yet described in published literature, software must be made available to editors/reviewers. We strongly encourage code deposition in a community repository (e.g. GitHub). See the Nature Research guidelines for submitting code & software for further information.

Data

Policy information about availability of data

All manuscripts must include a data availability statement. This statement should provide the following information, where applicable:

- Accession codes, unique identifiers, or web links for publicly available datasets
- A list of figures that have associated raw data
- A description of any restrictions on data availability

The datasets analyzed in this study archived on Figshare (http://doi.org/10.6084/m9.figshare.11300534). The source data underlying Figs 1-4, Supplementary Figs 2-4 and Supplementary Figs 6-11 are provided as a Source Data file.

Field-specific reporting

Please select the one below that is the best fit for your research. If you are not sure, read the appropriate sections before making your selection.

☐ Life sciences ☐ Behavioural & social sciences ☒ Ecological, evolutionary & environmental sciences

For a reference copy of the document with all sections, see nature.com/documents/nr-reporting-summary-flat.pdf

Ecological, evolutionary & environmental sciences study design

All studies must disclose on these points even when the disclosure is negative.

Study description	To identify the groups of natural enemies that may be responsible to community-wide conspecific density dependence , we manipulated the access of three groups of natural enemies, large herbivores (using fences), insect herbivores (using insecticide) and fungal pathogens (using fungicide), to seedlings in 180 1m × 1m quadrats in an old growth temperate forest in Northeast China. Further, we examined whether variation in the strength of conspecific density dependence among tree species is associated with two important plant traits: type of mycorrhizal association and shade tolerance.
Research sample	We established three 55 m × 50 m blocks, separated by at least 200 m within an old growth temperate forest. Each block was split into two equally-sized fenced and unfenced plots. Within each plot, thirty 1m × 1m seedling quadrats were equally divided and randomly allocated to each of the following treatments: fungicide (F), insecticide (I) and water (control of pesticide treatment; W). Across the 180 1 m × 1 m quadrats, 3929 individual seedlings recruited from 16 species in the censuses from 2015 to 2017.
Sampling strategy	We focused on the community-scale results, thus all 3929 individuals were tested as a whole.
Data collection	All woody plants less than 1 cm DBH were tagged, mapped and identified to species in all quadrats. In September of each year, we checked the status (survival/dead) of existing seedlings. In June 2017, we identified all adult trees (DBH > 5cm) within each block and recorded their species identity, DBH and distance to each quadrat within 20 m.
Timing and spatial scale	We calculated recruitment as the number of new seedlings > 1 cm tall in each quadrat in June each year. Seedling survival was checked in September, the late growing season in our temperate forest. Censuses were conducted across three years and across all 180 quadrats in three blocks.
Data exclusions	No data were excluded from the analyses.
Reproducibility	We conducted sensitivity analyses by randomly removing 25%, 50% and 75% of individuals of two dominant species (accounting for 74% seedlings) from the community data set, repeating this procedure 999 times. We found that our community-wide results were insensitive to removing random fractions of individuals of these two dominant species. Thus, we believe our results are robust and could be repeated in other temperate forests.
Randomization	In our experiment, all treatments were randomly allocated to each quadrat. Meanwhile, we considered quadrat and species identity as random intercepts and allowed the effect of census to vary among quadrats as a random effect.
Blinding	Blinding is not relevant to our study. We collected field data by recording the status, species identity, number of individuals, and DBH of woody plants, and did not exclude any data for analysis.

Did the study involve field work? ☒ Yes ☐ No

Field work, collection and transport

Field conditions	This study was conducted in an old-growth temperate forest. The climate is characterized by an annual mean temperature of 2.8 °C (-13.7- 19.6 °C) and average annual precipitation of ~700 mm, mostly as rain from June to September.
Location	This study was conducted within the Changbai Mountain National Nature Reserve in northeast China (42°23′ N, 128°05′ E, ~ 800 m a.s.l).
Access and import/export	We can access the study area freely, and do not need to get permission to collect data.
Disturbance	The study area is located in the Changbai Mountain Nature Reserve, and has been spared from severe distances for about 300 years.

Reporting for specific materials, systems and methods

We require information from authors about some types of materials, experimental systems and methods used in many studies. Here, indicate whether each material, system or method listed is relevant to your study. If you are not sure if a list item applies to your research, read the appropriate section before selecting a response.

Materials & experimental systems

n/a	Involved in the study
☒	☐ Antibodies
☒	☐ Eukaryotic cell lines
☒	☐ Palaeontology
☒	☐ Animals and other organisms
☒	☐ Human research participants
☒	☐ Clinical data

Methods

n/a	Involved in the study
☒	☐ ChIP-seq
☒	☐ Flow cytometry
☒	☐ MRI-based neuroimaging
